# “These people, you just guide them until they become these people”: learning to become a frequent indoor tanner

**DOI:** 10.1186/s40359-017-0181-4

**Published:** 2017-04-04

**Authors:** Jerod L. Stapleton, Benjamin F. Crabtree

**Affiliations:** 1 Rutgers, The State University of New Jersey, Rutgers Cancer Institute of New Jersey, 195 Little Albany Street, Room 5570, New Brunswick, NJ 08903 USA; 2 Rutgers, The State University of New Jersey, RWJ-Family Medicine-Research, Institute for Health, Health Care Policy and Aging Research (IFH), 112 Paterson Street, New Brunswick, NJ 08901 USA

**Keywords:** Ethnographic interview, Key informant, Indoor tanning, Melanoma prevention, Skin cancer prevention, Young adults

## Abstract

**Background:**

Many young women experiment with using indoor tanning beds with some becoming regular users. There is a dearth of research focused on factors related to the development of regular tanning. This study was designed to gain an in-depth understanding of the experiences of a regular indoor tanning bed user for the purpose of discovering working hypotheses related to the development of this behavior. The article thesis is that initial interactions with tanning salon employees transmit insider knowledge that serves to encourage the regular use of indoor tanning beyond experimentation.

**Methods:**

We used Spradley’s ethnographic interviewing technique to conduct six iterative interviews with a key informant who was an active indoor tanning bed user and former salon employee. The research was completed in the United States in 2015.

**Results:**

The informant described her experiences as a salon employee including her interactions with salon patrons. The informant was trained as a salon employee to talk about tanning as a complex process that requires multiple salon visits to achieve desired results and to develop rapport with salon patrons to be viewed as an important source of guidance and advice. In the informant’s experience, indoor tanning users who viewed tanning as a complex process and felt connected to salon employees were more receptive to purchasing larger amounts of bulk tanning sessions and committing to purchasing salon memberships.

**Conclusions:**

Findings provide insights into our understanding of the development of regular tanning behavior and we propose working hypotheses about this behavior to be examined in future research. There are also implications for policy makers to reduce excessive tanning behaviors including considering point-of-sale regulations that limit sales techniques of salon employees and pricing restrictions.

## Background

The use of artificial ultraviolet radiation-emitting indoor tanning beds is associated with an increased risk of skin cancer, including the deadly melanoma [1]. Despite the risks, nearly 10 million people use indoor tanning (IT) each year in the United States [2]. IT is most popular among young adult Caucasian females with nearly 1 in 3 reporting IT use in the past year and nearly 1 in 5 reporting regular use, defined as using IT 10 or more times in the past year [3]. Although any lifetime use of IT is associated with an increased risk of melanoma and other skin cancers, the risk is exponentially higher with regular use [4].

Much of the research related to behavioral reasons for IT has used survey methods to apply constructs from various health behavior theories (e.g., Theory of Planned Behavior [5] and Social Cognitive Theory [6]). Researchers have shown that appearance enhancement is the primary motivation for IT among most users [5–7]. IT users believe that tanning, or darkening one’s skin color through ultraviolet radiation exposure, leads to increased attractiveness and confidence in appearance and also believe that their peers use and approve of IT [6–9]. Exposure to beauty magazines is associated with positive tanning attitudes [10], which may lead IT users to believe that being tan is a defining characteristic of an “attractive” woman [7]. Many young women first use IT as high school students in the weeks leading up to certain events that represent rites of passage including school dances or birthdays [11, 12]. The popularity of IT, the shared view that a tan is attractiveness, and tanning narratives surrounding special events suggest tanning plays an important part of youth culture among young Caucasian women and explain why many experiment with IT. A subset of IT experimenters progress into prolonged or regular tanning, greatly increasing their risk of developing skin cancer, but there is a dearth of studies designed to identify factors underlying the development of regular IT. Our goal in conducting this study is to gain an in-depth understanding of the unique experience of a regular IT user for the purpose of discovering working hypotheses related to the development of this behavior.

This study is guided by the ethnographic perspective that discovery of cultural knowledge is a valuable first step in exploring understudied behaviors. Cultural knowledge is defined as the insider information that is shared among a group of people, learned through sociocultural experiences and interactions, and guides behavior [13]. The current research utilized a series of ethnographic interviews with a single key informant to gain insights into tanning culture by capturing the language and terms used when describing her experiences as a tanner [13, 14]. Our approach began with asking general, descriptive questions to avoid biasing the informant’s responses by using questions developed based on the interviewer’s assumptions or interests [14]. Information gained from early interviews guided the development of subsequent interviews and analyses to produce thematic summaries. The use of multiple ethnographic interviews provides the unique opportunity to gather information on an ongoing basis, get greater clarification and understanding, and check the interviewer’s understanding and interpretation with the informant [14, 15]. These interviews produce a rich description of the insider information that guides IT, which is utilized to formulate working hypotheses about the underpinnings of this behavior [16, 17].

As is common in the ethnographic interview discovery process [14, 16], the thesis of this paper emerged during the interview process. For most experimenters, IT first occurs at a tanning salon. Assuming that many tanners know little about IT prior to their first salon visit, their view of tanning is likely to be influenced by their encounters with salon employees. The thesis of this article is that the initial interactions between inexperienced salon patrons and salon employees serve to provide cultural knowledge and rules about IT that encourage continued use of IT beyond experimentation. This research is novel in describing such aspects of the salon employee-patron relationship.

## Method

### Participant and recruitment

We sough to identify a key informant who was knowledgeable about and active in the IT salon culture, had access to observations and perspectives not available to the researcher, and was willing to share her knowledge and experience [15]. Study eligibility requirements included 1) female gender, 2) between the ages of 18–25 years old, and 3) use of an IT device at least 10 times in the past 12 months. This IT criteria is commonly used in studies to identify high-risk tanners as it corresponds to a frequency of tanning well above rates associated with a greatly increased risk of melanoma [4, 18]. We targeted young adult female tanners because this group is most likely to engage in IT [18]. Approximately 1 in 5 young adult non-Hispanic white females engage in high-risk tanning with much lower rates among older females and males of all ages [3, 18] (except young gay and bisexual men [19]).

Recruitment consisted of posting study flyers on a large Northeastern United States University campus. The study purpose was described as attempting to better understand tanning behavior from the perspective of tanners. Interested participants emailed the interviewer who then scheduled a study eligibility screening phone call. The participant signed an informed consent form prior to the first interview and provided permission to audio-record the interviews. The Rutgers University Institutional Review Board approved the study.

### Research team and reflexivity

The first author holds a PhD and has conducted several qualitative data collections. The second author holds a PhD and is a widely recognized leader in the field of qualitative health research. The second author has mentored the first author in qualitative research and monitored the study. Both authors were faculty members of University academic departments at the time of study. Both researchers are male. The first author conducted all interviews. The authors worked together to conceptualize the study, develop the interview approach, and draft the first interview guide. The researchers and informant did not have a relationship prior to the study. Prior to the first interview, the interviewer briefly described to the informant his experience with studying IT using survey research and explained the purpose of the current study was to get a better understanding of IT by speaking with current tanners about their experiences. The interviewer described the ultimate goal of the research was to produce scientific reports. Per Spradley’s recommendations [14], the interviewer repeatedly stated that the informant was the expert and his goal was simply to learn about her IT experiences.

### Interview design

We followed the general principles outlined in Spradley’s *The Ethnographic Interview* [14] in conducting this key informant interview study and analysis. The general goals of the interviews were to: 1) have the informant describe her experiences as an IT user; 2) identify the language and terms used in these descriptions, and 3) use this information to create a description of the important insider information guiding tanning behavior. The first interview contained a variety of grand tour descriptive questions designed to encourage the participant to talk in a conversational way about her experience as a tanner using her typical language [15, 20]. For example, the following grand tour question was designed to have the informant talk about her IT: “Could you tell me all the things that typically happen when you go tanning, from when you get ready to go, to when you arrive and are tanning, until you are finished?”. The use of general, descriptive questions to begin the interview process allows participants to answer questions freely and helps to avoid the biasing of informants’ responses that can be caused by asking questions that reflect the interviewer’s interests [14]. Each audio-recorded interview was immediately transcribed and coded by the first author prior to the next interview in order to guide the development of subsequent interview scripts (see Interview Process and Analysis section). Over the period of repeated interviews (we had planned to use the typically recommended 5–6 interviews [15]), we slowly introduced new content based on prior interviews and the focus of the interviews shifted from eliciting descriptive information to utilizing questioning approaches designed for structured, thematic analysis and interpretation. All interviews lasted between 60 and 90 min and were held in closed small group study rooms in the University campus library. Only the interviewer and informant were present during interviews. The participant received a $30 gift card at the end of each interview.

### Interpretation

Of central concern to the rigor of qualitative research is ensuring the interviewer’s interpretations and study findings are accurate representations of informant’s experience [16, 17]. Several study design elements ensure rigor in our approach. First, the use of a series of interviews allows for multiple opportunities to explore insights not possible with one-time data collections. Second, analyses were conducted on an ongoing basis and subsequent interviews were always informed by prior interviews. Thus, we iteratively developed our interpretations and revised our analysis throughout the project. Third, the interviewer used a variety of techniques to check his ongoing understanding and interpretation [13, 14]. This included getting feedback and confirmation from the informant on working analyses, which served as a form of inter-rater reliability check. The study authors had email and in-person discussions during the study to generally review the ongoing analysis and to discuss the interview progression.

### Interview process and analysis

The analysis was primarily conducted by the first author in consultation with the informant following the guidelines outlined by Spradley in *The Ethnographic Interview* [14]. The overall goal of the analysis was to discover cultural themes derived from the data. This was accomplished in a series of building analytic steps. First, we asked broad, descriptive questions designed to elicit stories and descriptions containing important folk terms that captured insider cultural knowledge. The interviewer analyzed the transcript and field notes from the first interview for the purpose of identifying folk terms that may represent broader cultural categories called domains. For example, several different folk terms were identified that appeared to belong to a larger category of “Types of indoor tanning beds”. Domains are important to understand because they provide insights into the underlying order or structure of cultural knowledge. Second, these folk term lists were used to create structural questions for subsequent interviews that were designed to uncover domains by asking the informant to describe how various folk terms relate to each other or grouped together into broader cultural categories (e.g., “You mentioned ‘bronzer’ in your last interview. Is ‘bronzer’ a type of something?”). Information derived from structural questions was used to create a working list of identified domains.

Third, as we began to formulate possible domains, we introduced various contrast questions and exercises designed to discover the underlying features that defined and differentiated these domains. For example, “Tell me about the important differences between a bronzing bed and a melanin-building bed”. The fourth step was componential analysis, defined as creating comprehensive listings of identified domains along with detailed descriptions of the attributes and folk terms that defined each one. We began to create tables of various domain and componential analyses after the third interview. During the final three interviews, the informant was presented with various tables showing the working analyses and was asked to comment on their accuracy and clarify additional domain contrasts. We also utilized role-playing scenarios in which we asked the participant to recall her interactions within a tanning salon as an additional method of evaluating our analysis. In the sixth and final interview, the domains that form the basis of the themes presented in this article were confirmed and refined by the informant as a form of member checking [21]. At this time, we reached a point of data saturation as the informant had minimal clarifications on our themes and we felt that we had explored all relevant cultural categories that emerged during the interviews. We asked the informant to review the final manuscript as a final validity check and she confirmed the accuracy.

## Results

### Participant

We received email responses to our flyers by seven individuals. We were able to screen and schedule initial interviews with three participants. The choice to focus on the informant in this report was made because she had a wealth of unique expertise that we were able to deeply explore over the course of several interviews. A second participant was interviewed once as we uncovered she was an active indoor *sunless* tanner rather than *UV* tanner. A third participant was interviewed on three occasions at which time we felt that the knowledge gained from these interviews was incremental above that gained from interviews with the chosen informant.

The informant, Jessica (real name withheld), was 22 years old, self-identified as non-Hispanic White, and lived in the Northeastern U.S. We conducted a total of six interviews with her. The first five interviews were conducted in a 1-month span between June and July 2015 with the final interview in October 2015. She had first used her local tanning salon to prepare for her junior prom. She began a part-time job working at this salon after high school and continued throughout the next year. Jessica provided a depth of knowledge about many aspects of IT from her experience as a tanning salon employee, or salon *sales associate*, and her experience as a tanner in the years since leaving the job. She continues to use this local salon and tans two to three times per week during the winter and spring months. She is less likely to use IT during the summer, as she prefers to instead tan by sunbathing at a nearby beach.

### The tanning salon

Jessica’s salon is located near her home and is one of several in a small local chain. It is located within a strip mall that also houses a gym and a nail salon. The salon has a front desk near the entrance and a small row of chairs for waiting customers, or *guests*. Behind the desk is a hallway with several doors on either side leading to small, enclosed rooms that contain various types of tanning beds. A salon sales associate is stationed at the front desk and is responsible for greeting each *guest*, confirming payment for tanning, assigning them to the appropriate tanning bed/room, selling tanning lotions, and advising them on the beds. In her salon, the sales associate was in control of starting and timing the tanning bed sessions.

### Types of tanners and their reasons for tanning

**Fig. 1:**
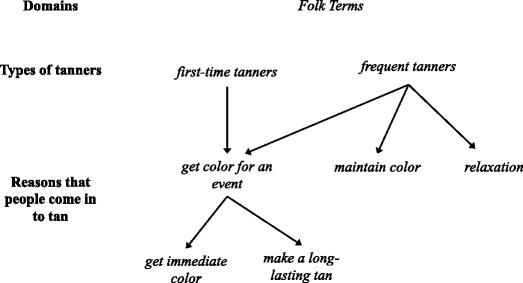
Indoor Tanner Types and Reasons for Use. Folk terms (denoted in italics) are the descriptions used by the informant and domains (denoted in bold) represent broader categories with multiple folk terms that emerged from the analyses. The two primary types of indoor tanners are first-time tanners and frequent tanners. Among the multiple reasons that people come in to tan, first time tanners are likely to want to get color for an event

A grand tour question in the first interview asked the informant to describe the other people she might see when at the salon. In recalling her experiences as both a tanner and a salon sales associate, Jessica described IT users both in terms of their varying knowledge and history of IT use (i.e., types of tanners) as well as their various reasons for tanning (Fig. 1). Speaking about IT users in these terms became a reoccurring theme and two important cultural domains emerged related to various types of tanners and various “reasons that people come in to tan”.

*First time tanners* have never before used or are inexperienced with IT and, as described later, require a great deal of information from salon employees:

“I’ve sat there for like half an hour, 45 min just explaining everything to them. They’re like ‘I didn’t know there was this much to know about tanning.’” (Interview 1)

Most *first-time tanners* come in to tan to *get color for an event. Get color* refers to tanning for the purpose of getting a darker or more tanned appearance. *Events* are certain celebrations or milestones during which “you want to look good because the spotlight’s on you” and include proms, weddings, graduations, and birthdays. For these events, the focus is on getting *immediate color* to quickly get a tan for the upcoming event. A *first time tanner* might say:

“‘I need to be tan for my wedding’… ‘I just want a glow. I didn’t want to be the same color as my dress.’” (Interview 2)

Other *events* include upcoming vacations in sunny locations or the weeks prior to summer. For these *events*, the focus is to *make a long-lasting tan* by obtaining a *base tan* with IT prior to the event. Participants with a *base tan* believe they will be less concerned about getting sunburned on their vacation and will use the prolonged sun exposure during vacation or the summer to build a tan rather than rely on IT.

*Frequent tanners* are at the salon often enough to be recognizable to sales associates and make up an estimated 80% of the salon clientele. Salon associates often learn personal information and have conversations with *frequent tanners.*

“You get to know them and they’re always interested in who’s working… you build relationships with the people and they end up, I guess, trusting you with what you’re doing with them in the beds.” (Interview 1)

*Frequent tanners* tend to fall into one of two subcategories. *Consistent* frequent tanners have a set schedule and tan on certain days each week. This schedule often revolves around times they are near the salon or after they finish their workday or gym workout. *Consistent* tanners use the same type of tanning bed for a similar amount of time each session. *Educated* frequent tanners have a deeper understanding of tanning and tend to purposefully vary their tanning to more closely match the patterns recommended by the salon for maximum tanning results. The tanning behavior of *educated* tanners is less scheduled compared to *consistent* tanners and they are more likely to go tanning when *they feel like it*. Unlike *consistent* tanners who are likely to maintain a certain level of tan given their consistency in exposure, *educated* tanners are more likely to go for periods of several days, weeks, or even months between regular IT use which results in a less consistent *color*. Jessica considered herself to be an *educated* tanner, described tanning as *work*, and actively monitored her tan level and adjusted her IT to achieve her desired results. *Educated* tanners sometimes experience periods of time when they do not feel motivated to go to the tanning salon, particularly when they have not been in a while and their tan begins to *fade*.

“I always get into those phases though. I think it happens with everyone. Some people I wouldn’t see them there for a while and then I’d see them there every other day. You’re just kind of like I have to get back into it… Once I go and I start getting color I’m there every other day. It’s just getting me to go, that’s the problem… There are some months actually where I haven’t gone at all but then there are some months where I’m there 3 times a week…” (Interview 6)

*Frequent* tanners*,* like *first time* tanners, are motivated to tan in order to *get color* prior to events but their primary reason for tanning differs in their desire to tan on a more regular basis in order to *maintain color*.

“[They are] the people with no specific purpose. No agenda. They just want to be tanned…It’s not for a specific reason.” (Interview 6)

Some *frequent tanners* perceive an added benefit of IT related to *relaxation*.

“You’ll have the people who…they turn it into whatever they want. I mean, they don’t always necessarily come for the color… [they] come in for that 20 min of peace…they come in, they go right to sleep, they know they’re going to go right to sleep. They take a nap.” (Interview 2)

### Educating guests on the tan process

In responding to grand tour questions about the experience of being in a salon, Jessica talked at length about interactions between the sales associates and guests before and after IT sessions. A theme of these interactions that began to emerge in the first interview related to the sales associate *educating guests* about the *tan process*. The *education* of *first time* tanners was an important focus of sales associates and often occurred as part of a salon tour. During a typical tour, a sales associate shows the tanner the salon, *explains* the different types of available tanning beds and lotions, and provides information about the *tan process.*

“…the first thing that we’re instructed to do as soon as somebody new walks in is…give them a tour of the facilities… You stop in every single room… You show them the bed, explain what it does… You’re basically just educating them when they first come in which would help them decide what is best for them and what they’re looking for.” (Interview 3)

*Educating guests* on the process of *building a great looking tan* involved explaining two fundamental processes of tanning: *getting color* and *building tolerance.* Further, the purpose of the various types of tanning beds were described relative to their ability to either produce *color* or *build tolerance* with these distinctions attributed to differences in the skin tan response to different types of bulbs with various types of UV emissions. Tanners interested in getting a one-time tan for an event or maintaining a constant tan could simply use beds and lotions designed specifically for enhancing skin *color*. The informant referred to this type of bed as a *bronzing bed* or more commonly by its manufacture’s product name and stated that *bronzing beds* were less likely to cause sunburns compared to other types of beds.

“The bronzing one has the least potential to burn you and that’s actually the more expensive bed and the bigger bed because that’s the one that actually gives you the color… If they have a special when you first sign up, it’s usually something like 7 days in any bed, that’s the one that people go into just because they come to tan and they want to see immediate color and that won’t burn you…” (Interview 1)

Once a tanner attains her desired *color*, she has the option to *maintain* her *color* by keeping with a consistent tanning pattern in a *bronzing bed*. However, tanners who desire to *build their color* to an even deeper tan face a potential problem because using the same bronzing bed repetitively can lead to a color *plateau.* A *plateau* describes the point at which a particular bed can no longer make tanners darker than their current color.

“You can go in the bronzing bed probably 4 times in a row and you won’t see any difference. You could go in there 10 days straight and you won’t see any difference, you plateau.” (Interview 1)

Tanners are told that if a tan plateau is reached they must use a different type of bed to further increase their tan level. The *melanin-building* bed is designed less for *building color* but for the purpose of *building tolerance*, defined by the informant as “how much [UV exposure] your skin can stand without burning”. *Melanin-building* beds produce less color after a tanning session compared to *bronzing* beds but the salon claimed they build base skin melanin that can be later tanned in the *bronzing* beds. Guests are warned that there is a greater potential of burning in a *melanin-building* bed compared to a *bronzing* bed. The process of *building tolerance* involves *starting off low* with a small amount of time in the *melanin-building* bed (relative to the manufacture’s maximum recommended exposure time) to avoid burning and *bumping up* the number of minutes in the bed with an ultimate goal of reaching the *max time* allowed in the bed.

“So when you go into the melanin bed, you don’t really see much difference and it does have the most potential to burn you so that’s usually the one you’re not going to pass more than 2 min at a time, starting low. That one brings about the melanin in your skin so that when you go into the bronzing bed, that bed can then bronze that melanin so that you are no longer plateaued.” (Interview 1)

**Fig. 2:**
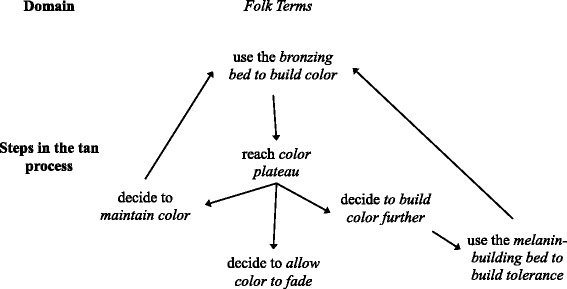
The Tan Process. The tan process involves variable use of tanning beds for the purposes of both building immediate color as well as building tolerance to allow for the eventual development of an increasingly darker tan

By framing tanning as a *process*, salon sales associates convey the message that optimal tanning occurs with frequent visits and *rotating* with different beds and lotions rather than occasionally using a single tanning bed (Fig. 2).

Once someone understands and experiences this process, they can determine when they reach a *color plateau* after multiple uses of the *bronzing* bed and then decide whether to *maintain* the color by continuing to return to the *bronzing bed*, stop tanning and allow their tan to *fade*, or *build color further. Building color* necessitates *rotating* to the *melanin-building* bed to *build their tolerance/melanin* and then returning to use a *bronzing* bed to *maximize* their tan by tanning the newly acquired melanin. Once a new *plateau* is reached at a darker tan level, tanners once again decide whether to *maintain* the color, *build it* further, or allow it to *fade* by taking a few days off from the tanning salon. The following summation of the *tan process* was provided by Jessica when asked to role play what she might recommend to a *first time* tanner who wanted to *get some color* in the month prior to his wedding.

“I would probably say, You have a month so go into the [bronzing bed: informant used the manufacturer’s product name which is withheld], start building your color within your first three times and then at that point you would rotate to the [melanin building bed: product name withheld]…and somewhere in the middle of the month, throw in a few more [bronzing bed sessions], still rotate, somewhere at the end of the month or pretty much your last three times of coming, you want to go out with a bang, your three [bronzing bed] sessions and that way you would not only maintain the color that you started in the beginning but you would build on it, because all of that rotating is building the melanin that the [bronzing bed] is going to tan. Then doing the same thing and at the end, the last three times, you get all that color.” (Interview 5)

### Building rapport

Another important task for salon associates was to develop *rapport* with guests which for *first time* tanners involved determining their reasons for coming in to the salon or their *needs*:

“…you are personalizing it [tanning] and customizing it to them. So you need to know what their needs are, that’s your rapport with them… your rapport is kind of like getting to know them… sort of saying like ‘What are you tanning for?’. ‘Oh, for like a wedding?’. And then that’s when you’d be like ‘Oh, whose wedding?’… it’s not just about tanning so that’s where the rapport comes in…they feel it’s more personal for them which also makes the sales pitch easier because at least they were comfortable with you.” (Interview 6)

The assessment of *needs* often includes asking about *guests’ tan goals*, described as their desired skin tone or shade to achieve from tanning. In determining *tan goals*, Jessica would often ask guests about their desired tan relative to her own.

“I’ll use my own skin as a reference… ‘Do you want to be my color, darker than me, lighter than me?’. And that just basically helps me plan out kinda how long they need to be coming. And, how often they need to be coming. If you don’t want to me darker than me, you don’t have to come in as often … So I could tell you to come in every 3 or 4 days because you’re not trying to get that dark. But you still want to be progressing because you don’t want to get to your color and stop and have 2 weeks before your wedding and fade all of that time because you just, color does fade if you don’t maintain it.” (Interview 2)

The assessment of *needs* and *tan goals* allows the associate to develop a personalized *plan* for each *first time* tanner. The tan *plan* can involve detailed guidance about which beds to use and incorporates information from the tan tour. Sales associates claim that following the *plan* will help to keep guests safe from burning by providing more control over their exposure.

“I’ve kinda created almost like a calendar or a timeline. Like you know, ‘Come in every 2 days, start with this bed, see how you react. These are your options based off of what that does to you’. Kinda just progress… And then, they’re like ‘Oh, that was so helpful!’” (Interview 2)

Once *first time* tanners have some experience, and to a lesser extent with *frequent* tanners, the *rapport* process evolves into providing ongoing advice and support related to tanning. This may include helping guests determine which bed they should use, how long they should tan, and advice about using tanning lotions. *Rapport* also relates to getting feedback about their tanning experiences after an IT session to ensure that they had a good experience.

“After they’ve come in a few times…I would say ‘What do you want to do today?’ That’s usually something that came out of my mouth constantly… if I was the one that made the plan for them I you know would kind of ask if they’re keeping on track or what they did last… And at that point some people remember. They’re like ‘Well today I should do this?’ and the plan’s not always perfect. Today you should do this but how did you react to it, that bed last time. So you adjust as you go…You’re kind of guiding them because they don’t always know that you don’t have to stick to the exact plan.” (Interview 6)

### Working out the pricing

There are multiple options for paying for tanning salon services. The first option is to pay for a *single session* at a time. Another are packages that allow guests to purchase a bulk amount of tan credits to pay for the cost of the session at a price that is cheaper than paying for a *single session* (for example, purchasing 50 tan credits for the price of 40 U.S. dollars). *Memberships* allow for unlimited use of certain types of tanning beds that are sold on a monthly basis with a single fixed price. *Memberships* are often sold as attractive options to guests because the per-price fee for each session ends up being much less than purchasing a comparable amount of tanning sessions using packages or single sessions. In Jessica’s experience, a guest who tans multiple times per week would end up paying about half as much per tanning session with a *membership* compared to paying for *single sessions*. *Memberships* often include up-front sign-up fees and automatic re-enrollment via credit card charges until a guest cancels the membership, which both may promote guests sticking with packages for an extended period of time.

Jessica used the terms *sales pitch* and *work out the pricing* when describing how sales associates advise guests on how to spend their money once at the salon. In deciding what to purchase, the guest has to choose both the type of tanning bed and also their payment option. An important purpose of the salon associate *educating guests* about the *tan process* and working to develop *rapport* and a *tan plan* is to make it easier to sell tanning *memberships* and *packages* rather than *single sessions*.

“You explain everything to them during the tour just to see if there’s really you know no questions when it comes to a particular plan for them. It’s kind of like ‘Ok, well you’re going to need this bed this day and then like a few days later you’re going to switch to this bed’. And they’re going to know why because you just explained it to them. And then they’re also more open to the packages that you give them because they understand that they need to be doing more than one bed. So it’s not like ‘Oh my god, I’m just going to do the cheaper bed’ because I just explained to them why it’s not going to help you.” (Interview 6)

Jessica shared that although she typically only *pitched* memberships to guests who clearly planned to tan for multiple months at a time, salon management encouraged associates to sell as many memberships as possible and only sell single sessions as a last resort. Several aspects of the memberships made them difficult to cancel and served to keep newly enrolled guests returning. For example, her salon’s policy was that memberships could not be canceled within the first 30 days, which effectively automatically enrolled those who purchased new members into at least a 2-month commitment. Some salons also established cancellation policies that involved writing letters or making phone calls to cancel memberships. Further, guests who had canceled their monthly memberships in the past were required to pay an additional enrollment fee each time they signed up for a new membership. Instead of canceling their memberships in times of non-use, which would incur new enrollment fees, users could *freeze* their membership by paying a small monthly deactivating fee and reactivating it at a later date. Jessica described how being enrolled in a membership may also pressure IT users into going tanning even when they might not feel the desire to go so they would feel like they were *getting their monies worth*.

“… today I did not feel like it so I did not go tanning but I wanted to yesterday and then today I was like I don’t really feel like it so I didn’t go. But I was too lazy to go so I guess the ones that do come frequently are not lazy. Probably want their monies worth. **[Interviewer question: Their monies worth?]** Yeah, I tell myself, I haven’t gone in September and it’s September 17 so I paid for the month and I haven’t gone for 17 days so I’m like I should really get my monies worth so then yesterday I’m like ‘Ok I’m going to go’.” (Interview 6)

S*pecials* and *deals* describe a variety of sales and discounts designed to either attract *first time* tanners by discounting single session pricing surrounding prom season or temporarily reducing the price of memberships. A less obvious benefit of these specials is they also encourage frequent IT users who have not tanned for a short time or may have frozen memberships to return to the salon. During our final interview, Jessica said that she had not been tanning for several weeks but had maintained her account in a reduced price or frozen state. In response to a question of whether she planned to tan again in the near future:

“Probably…usually what gets me to go back is like a sale. So that becomes my motivation, the motivation that I’m missing right now…**[Interviewer: ‘So with a sale you’ll be able to get a better bed for…’]** Yea it’s cheaper because the upgrade price is usually lowered or they’ll have some type of package or like 7 days in any bed for however much money. And once you do that and they start to see color then they’re like ‘Ok, I should probably keep going to keep the color I just paid for’. And then it starts up again. That’s how they reel you in.” (Interview 6).

## Discussion

The goal of this study was to explore the experiences and insider cultural knowledge of a young woman engaged in regular IT. The ethnographic interviewing approach provides a detailed understanding of the insider knowledge and contextual factors related to IT and reduces potential bias introduced by researchers using more structured data collection approaches like surveys. The study thesis was that interactions between IT patrons and tanning salon employees serve to encourage continued use of IT. The informant described educating first time tanners about the tan process which involves tanning for the purpose of both building immediate color as well as building tolerance (i.e., tanning in a bed that produces less immediate color but allows for greater later development of color). As a salon employee, she also sought to build rapport with patrons by encouraging them to identify their tanning goal and develop a tanning plan. The informant believed the process of educating guests and building rapport made it more likely that patrons would be willing to purchase expensive salon memberships that encourage repeated tanning.

### Emerging hypotheses

The informant’s description of her training as a salon employee and subsequent interactions with salon patrons represents a previously unexplored relationship. For young women experimenting with IT, their view of tanning is likely to be influenced by their initial encounters with salon employees. An emerging hypothesis is that the IT industry has a unique marketing avenue through the direct influence exerted by tanning salon employees on tanners. This influence may involve training salon employees to use sales techniques and give guidance to novice, experimenting tanners designed to result in extended use. Contrast the role of the salon employee to that of a clerk at a convenience store who simply serves as a cashier for purchasing cigarettes or unhealthy food options. Public health IT industry research has followed the lead of tobacco control research to document various types of advertisements and marketing strategies (e.g., [22]). However, this approach appears to overlook the unique, interpersonal aspect of IT marketing by salon employees. The few existing studies of tanning salon operators have primarily focused on whether salons employees are compliant with federal or state regulations related to exposure limits [23] or restrictions on access to tanning beds among minors [24]. Other researchers have examined whether tanners receive relevant warning and safety guidelines at salons [25]. Our findings support the need for future research to utilize representative samples to systematically capture the type and prevalence of information routinely shared by salon employees as well as to examine the association with uptake of regular IT.

Studies have used expectancy-value theories (e.g., Theory of Planned Behavior) to identify beliefs and attitudes associated with a history of IT use (e.g., [5–7]). However, the factors associated with the uptake and maintenance of regular IT have received little attention and general tanning beliefs (e.g., the belief that tanning is attractive) may be less helpful in explaining why some experimenters progress to regular, habitual IT. The current findings provide insights into the central role an unexplored contextual factor, the tanning salon experience, may play in the development of regular IT. The description of tanning as a complex process that requires multiple salon visits to achieve desired results provides an explicit set of insider information or rules for tanners to follow if they want to enhance their attractiveness with tanning. Self-regulation theories, which have not been applied to tanning, posit that in order for a habit to take hold individuals must develop mental models or plans for how to utilize habitual behaviors to achieve desired goals [26]. Thus, an additional emerging hypothesis is that the description of tanning as a process by salon employees and their ongoing guidance serves to build a mental model of the routines and rules of tanning for inexperienced tanners that may lead to habitual use to maintain appearance goals. Future survey research should seek to uncover the goals, plans, or rules of regular IT users to better understand the habitual nature of IT.

### Supportive evidence for findings

The purpose of our ethnographic approach was to elicit hypotheses about the development of regular IT based on an in-depth understanding of a tanner’s experiences rather than produce generalizable knowledge of the experiences of many users. One approach to supporting the external validity of such findings is to use modal comparisons, defined as describing how typical the informant’s experience is likely to be compared to similar others [17]. Tanning salons are often owned by independent operators or within small, regional chains and there are no uniform federal regulations that would require a standardized training for employees [25]. Thus, salon employee training and incentivizing is likely to vary depending on salon ownership. Several companies do provide standardized in-person and online training for IT salon employees that cover topics ranging from basic safety and sales procedures. These trainings appear to be heavily encouraged by the IT trade industry groups, who have claimed the wide utilization of such trainings are an effective method of industry self-regulation [25]. A 2014 poll of salon owners reported on an industry website claimed that 79% of tanning salon employees receive at least 10 h of training [27]. Further, salon safety trainings are required of all tanning salon employees in five states [28]. Training experiences such as those described by the informant are likely to be fairly common.

There is a dearth of empirical, peer-reviewed studies related to salon operator training or communications with salon patrons. In an attempt to corroborate our findings, we identified and reviewed several recent articles from IT industry publications and websites as well as materials from IT salon training programs. Many of these sources highlight the importance of educating clients. For example, promotional materials for an industry-leading employee training program focus on the importance of training on the “interaction between employees and customers” [29]. Recent articles published in an online IT industry website stress the importance of teaching sales techniques for salon employees [30] as well as providing salon tours to give patrons the “attention and education they need” to maximize sales [31]. The National Tanning Training Institute (NTTI), which claims to have trained over 20,000 salon operators [28], has a basic certification course that includes a section labeled “tanning process”. The NTTI also has a salon operations and procedures course that recommends salon owners train employees on how to talk to patrons including learning suggested answers to frequently asked questions [32].

In describing the importance of educating clients, the theme of building rapport is also present. The February 2017 issue of an industry magazine profiles a successful salon owner [33]. In stressing the importance of educating patrons, the salon owner is quoted as saying: “We thrive on education. It’s important to know what you’re doing and talking about instead of just letting the customer do what they want. I like educating anyone on anything because I can help them making [sic] better decisions” [33]. The NTTI’s operations manual suggests that new tanners receive special treatment, are shown around the salon, and receive an explanation of the equipment and services [31]. The manual emphasizes that spending time with the customer and finding out what they want and need are essential to effective selling. Another online article focused on salon customer retention stresses the importance of knowing clients well and “….finding out their tanning goals” [34]. Another online article titled “How Pushy is TOO Pushy” states: “They [top salespeople] create a vision and engage their customers in owning the vision. They just don’t sell…they guide them [salon patrons] into products or services that will aid in achieving their ultimate [tanning] goals.” [35].

The informant’s description of tanning beds types and the tanning process are consistent with what a tanner might find with a web search using terms such as “melanin building bed”. For example, a tanning lamp sales website contrasts “regular tanning lamps” with “bronzing lamps” on their ability to impact the skin tanning response [36]. A tanning salon website assures potential customers that operators will help customers “…get on the path to the tan you’ve been dreaming of” which starts with using basic beds to accomplish a “tanning goal” and mixing up tanning by adding sessions in a bronzing beds [37]. Another tanning tips website uses similar terminology when advising on how to adjust tanning after reaching a “plateau” and describing the optimal tanning frequency to avoid “fading tans” [38]. Another tanning salon webpage provides detailed explanations of the differences in types of UV rays emitted by “burning” vs. “non-burning beds” and explains that a tanning “plateau” can be avoided by switching bed types [39].

### Implications

Our results have implications for IT policy. IT policy efforts have largely focused on states enacting restrictions of access to IT by minors [40]. A 2015 proposal by the U.S. Food and Drug Administration sought to enact a nationwide ban of IT among those under the age of 18 [41]. These efforts are intended to reduce the amount of consumers, as was the result of bans of minors from using other unhealthy behaviors (e.g., restrictions on tobacco or alcohol) [42]. The U.S. Federal Trade Commission and other governmental agencies have also enacted restrictions on IT industry advertisements that have contained questionable claims of health benefits or are dismissive of risks [43]. However, these policy efforts have not addressed greater regulation of IT at the point-of-purchase other than requiring the provision of standardized risk warnings [44]. Restrictions on advertisements could be extended to point-of-sale such that the information provided by salon employees would follow established protocols designed to reduce unnecessary exposure rather than providing information and using sales techniques that seek to maximize number of the salon visits. Such restrictions may have the benefit of reducing the number of first time tanners who transition to become regular users, particularly important from a public health perspective as risk for skin cancer and melanoma rises exponentially with regular use [4].

The informant’s salon used sales, package deals, and other price discounts to encourage the purchase of bulk tanning sessions, which is consistent with research that shows salon patrons often receive such marketing directly from tanning salons [45]. The informant described how selling IT sessions in bulk was often effective at bringing first time tanners back to the salon for multiple times with the hope of turning them into repeat customers. This suggests a possible benefit of setting pricing controls on the sales of salon services. If patrons were forced to buy single sessions at the salon, it may reduce the likelihood that patrons will feel pressured to get their “monies worth” and return to the salon to utilize additional sessions purchased as memberships or as a part of a larger package. Limiting the ability of salons to have bulk sales around prom season may effectively reduce the total number of sessions that a first time tanner is likely to engage in.

### Study strengths and limitations

A strength of the ethnographic interview approach lies in the unique ability to gain deeper insights that are difficult to obtain with single interviews. The use of knowledge checks with the informant and multiple interviews as well as adherence to recommended guidelines for comprehensive reporting of qualitative research (i.e., the consolidated criteria for reporting qualitative research (COREQ)) [45] represent study strengths that bolster internal validity [17]. The goal of describing a detailed, personal perspective is incongruent with establishing constructs that are generally true of many different people (i.e., generalizability), which requires careful sampling of many individuals [16, 17]. Generalizations drawn from this research should accordingly be viewed as emerging hypotheses rather than conclusions about universal principles. These findings may not be applicable to typical tanners’ experiences at other salons and the hypotheses generated from this study should be tested in larger, more representative samples. Another potential limitation is that the interviewer was male although the use of multiple interviews allowed the interviewer to build a strong rapport with the informant.

## Conclusion

This article provides novel insights into the transmission of knowledge between a tanning salon employee and salon patrons. Our findings suggest employees may be trained to create an understanding of tanning as a complex process among patrons that involves repeated visits to the tanning salon. This may have the effect of encouraging first time tanners who are experimenting with tanning to transition to more frequent tanning. This research is suggestive of the need for future research to better understand the transition from first time to frequent IT use as well as suggests avenues for enacting policy efforts that may reduce IT use.

## Abbreviations

IT: Indoor tanning
